# Are *Toxoplasma*-infected subjects more attractive, symmetrical, or healthier than non-infected ones? Evidence from subjective and objective measurements

**DOI:** 10.7717/peerj.13122

**Published:** 2022-03-25

**Authors:** Javier I. Borráz-León, Markus J. Rantala, Indrikis A. Krams, Ana Lilia Cerda-Molina, Jorge Contreras-Garduño

**Affiliations:** 1 Department of Biology, University of Turku, Turku, Finland; 2 Department of Biotechnology, Daugavpils University, Daugavpils, Latvia; 3 Institute of Ecology and Earth Sciences, University of Tartu, Tartu, Estonia; 4 Department of Zoology and Animal Ecology, University of Latvia, Riga, Latvia; 5 Department of Ethology, National Institute of Psychiatry “Ramón de la Fuente Muñiz”, Mexico City, Mexico; 6 Evolutionary Ecology Lab, National Autonomous University of Mexico, Morelia, Mexico

**Keywords:** *Toxoplasma gondii*, Parasites, Attractiveness, Health, Fluctuating asymmetry

## Abstract

**Background:**

Parasites are among the main factors that negatively impact the health and reproductive success of organisms. However, if parasites diminish a host’s health and attractiveness to such an extent that finding a mate becomes almost impossible, the parasite would decrease its odds of reproducing and passing to the next generation. There is evidence that *Toxoplasma gondii* (*T. gondii*) manipulates phenotypic characteristics of its intermediate hosts to increase its spread. However, whether *T. gondii* manipulates phenotypic characteristics in humans remains poorly studied. Therefore, the present research had two main aims: (1) To compare traits associated with health and parasite resistance in *Toxoplasma*-infected and non-infected subjects. (2) To investigate whether other people perceive differences in attractiveness and health between *Toxoplasma*-infected and non-infected subjects of both sexes.

**Methods:**

For the first aim, *Toxoplasma*-infected (*n* = 35) and non-infected subjects (*n* = 178) were compared for self-perceived attractiveness, number of sexual partners, number of minor ailments, body mass index, mate value, handgrip strength, facial fluctuating asymmetry, and facial width-to-height ratio. For the second aim, an independent group of 205 raters (59 men and 146 women) evaluated the attractiveness and perceived health of facial pictures of *Toxoplasma*-infected and non-infected subjects.

**Results:**

First, we found that infected men had lower facial fluctuating asymmetry whereas infected women had lower body mass, lower body mass index, a tendency for lower facial fluctuating asymmetry, higher self-perceived attractiveness, and a higher number of sexual partners than non-infected ones. Then, we found that infected men and women were rated as more attractive and healthier than non-infected ones.

**Conclusions:**

Our results suggest that some sexually transmitted parasites, such as *T. gondii*, may produce changes in the appearance and behavior of the human host, either as a by-product of the infection or as the result of the manipulation of the parasite to increase its spread to new hosts. Taken together, these results lay the foundation for future research on the manipulation of the human host by sexually transmitted pathogens and parasites.

## Introduction

Successful mate choice implies finding the “best possible mate”, in terms of biological quality (*e.g*., good genes, reproductive potential, and health) and behavioral quality (*e.g*., resource acquisition and parental investment) (*e.g*., Trivers, 1972; Geary, Vigil & Byrd-Craven, 2004; Luoto, 2019; Garza & Byrd-Craven, 2021). Therefore, selection favors mechanisms that allow organisms to identify and prefer phenotypic characteristics associated with good genes, strength, and health (Rantala et al., 2012) and, at the same time, to avoid those related to a poor genetic condition, susceptibility to disease, or poor health (Borráz-León et al., 2014; Curtis, 2014; Van Leeuwen & Petersen, 2018). Preferences for these traits may vary according to socioecological factors such as sociosexual orientation and socioeconomic position, as well as to individuals’ characteristics such as health status, personality, self-perceived attractiveness, or mate value (Pawlowski & Jasienska, 2008; Little, Jones & DeBruine, 2011; Kočnar, Saribay & Kleisner, 2019).

Among the factors that negatively impact the expression of both biological and behavioral traits are the presence of parasites (*e.g*., Zuk, 1992; Møller, 1990). Parasites can inflict physiological and energetic costs to their hosts (Luong, Horn & Brophy, 2017). For example, parasitized males exhibit impairments in the development of secondary sexual characteristics and physical strength, traits that are indicative of good health (Møller, 1990; Folstad & Karter, 1992). Therefore, parasitized organisms usually do not display conspicuous sexual characteristics and are competitively inferior, which ultimately diminishes their odds to mate and reproduce (Zuk, 1992; Jacobs & Zuk, 2012). However, if parasites diminish a host’s attractiveness and health to such an extent that finding a potential mate becomes almost impossible and survival is heavily comprised, parasites can decrease their own odds to reproduce and pass to the next generation, especially if the parasites’ route includes sexual transmission. Thus, host and parasites may have co-evolved strategies for living in equilibrium by placing constant demand on each other for adaptations and counter-adaptations (Dass et al., 2011). One of the strategies used by parasites that is of interest in the fields of evolutionary biology, ecology (*e.g*., Thomas, Adamo & Moore, 2005), and more recently, in evolutionary psychiatry (*e.g*., Del Giudice, 2019; Borráz-León et al., 2021a), is host manipulation, which occurs when a parasite increases its own transmission rate by altering host behavior, appearance, morphology, and/or physiology (Moore, 2002; Thomas, Adamo & Moore, 2005; Poulin, 2010).

Among the parasites that can produce changes in the aforementioned characteristics is *Toxoplasma gondii* (Webster, 2001; Flegr, 2013). *T. gondii* is a neurotropic intracellular protozoan parasite with domestic cat and other felids as definitive hosts and with a broad spectrum of intermediate warm-blood animal hosts, including human beings (Flegr, 2007; 2013). Since *T. gondii* must reach its definitive host to reproduce sexually, it changes the behavior of infected rats by reversing the innate aversion to cat odor into an attraction toward it, increasing the odds of being eaten by a cat (Berdoy, Webster & Macdonald, 2000). Similar changes have also been described for infected chimpanzees (Poirotte et al., 2016), infected hyenas (Gering et al., 2021), and infected humans (Flegr et al., 2011). Although the neurobiological mechanisms by which this parasite modify the behavior of its intermediate hosts are not completely known, previous evidence suggests that *T. gondii* infection may suppress the neural activity of limbic areas that modulate the innate defensive behavior whereas increases activity in nearby limbic areas that modulate sexual attraction in response to cat odors (House, Vyas & Sapolsky, 2011). Moreover, studies with rodents have proposed that these effects may be mediated by brain neurotransmitters and sex and stress hormones and their impact on the brain and behavior. For example, it has been shown that *T. gondii* can directly enhance brain serotoninergic and dopaminergic activity in its host through alterations in gene expression (Prandovszky et al., 2011; Xiao et al., 2014). Other studies have also associated *Toxoplasma* infection with dysregulation in gamma-aminobutyric acid (GABA), glutamate, and serotonin levels in rodents (Fuks et al., 2012; David et al., 2016; Mahmoud, Fereig & Nishikawa, 2017). The areas of the brain that are particularly susceptible to changes in these neurotransmitters are the nucleus accumbens and ventral tegmental area, which receive projections of the limbic system, including the amygdala (Haber & Fudge, 1997). Thus, altered neurotransmitter activity in these areas may be responsible for the emotional, motivational, cognitive, and behavioral changes observed in *Toxoplasma*-infected individuals (see Tyebji et al., 2019). Alternative hypotheses suggest that the behavioral and psychological alterations observed in *Toxoplasma*-infected individuals could be either a side-effect of lesions in certain brain areas produced by random allocation of the *Toxoplasma* cysts, or the result of the body’s reaction to the parasite infection (*e.g*., a prolonged release of cytokines and chronic inflammation) rather than by the parasite itself (Del Giudice, 2019; Flegr & Horáček, 2019). However, given that the behavioral changes of *Toxoplasma*-infected organisms can increase the risk of being caught by felines (*e.g*., lower reaction times, attraction to cat urine) (Flegr, 2013; Flegr & Horáček, 2019), the neurobiological and behavioral alterations produced by *T. gondii* in its intermediate hosts have been mainly interpreted as an evolutionary adaptation of the parasite to complete its life-cycle within its definite hosts and/or to spread to new bodies (Poirotte et al., 2016; Brüne, 2019; Borráz-León et al., 2021a).

Even though humans are no longer common prey of big felines, it is possible that *T. gondii* manipulated hominid behavior in the past, thus making our ancestors easier targets for big cats (Webster, 2001; Flegr, 2013). Some previous studies have suggested that some of the phenotypic changes associated with *T. gondii* infection might, at the same time, offer some indirect advantages to its hosts (Dass et al., 2011; Borráz-León et al., 2021a) and represent transmission-related benefits for *T. gondii* (Brüne, 2019; Del Giudice, 2019). For example, in one study, *Toxoplasma*-infected male rats were perceived as more sexually attractive and were preferred as sexual partners by non-infected females (Dass et al., 2011). Although females’ aversion to parasitized males may have evolved to avoid direct infection during mating and to minimize the chances to produce offspring with low heritable parasitic resistance (Hamilton & Zuk, 1982; Able, 1996; Van Leeuwen & Petersen, 2018), parasites may have developed counterstrategies to manipulate host appearance and behavior to overcome this typical female aversion (Dass et al., 2011).

Although this hypothesis has not been directly tested in humans, previous research has shown that *Toxoplasma*-infected men are, in average, 3-cm taller, and their faces are rated as more masculine and more dominant by females (Flegr et al., 2005; Hodková et al., 2007). Moreover, several independent studies have consistently shown that *Toxoplasma*-infected men have higher testosterone levels than non-infected ones (*e.g*., Flegr, Lindová & Kodym, 2008; Zouei et al., 2018; Borráz-León et al., 2021a). Thus, *T. gondii* may benefit by promoting the expression of testosterone-dependent traits in men, which normally provide information on different aspects of men’s quality (*e.g*., Windhager, Schaefer & Fink, 2011), thereby increasing their mating success (Rantala et al., 2012). A similar but opposite effect would be expected for women, such that *Toxoplasma*-infected women might display more feminine physical traits and have lower testosterone levels than non-infected women (Flegr, Lindová & Kodym, 2008; but see Borráz-León et al., 2021a). However, it is important to mention that research in the Czech Republic failed to detect significant differences between *Toxoplasma*-infected and non-infected subjects in other physical traits such as body mass index (BMI), waist-to-hip ratio, and body fluctuating asymmetry (FA) (see Flegr et al., 2005), highlighting the need to carry out similar studies in other populations.

Besides physical and physiological traits, there is some evidence for changes in behavioral and personality traits in *Toxoplasma*-infected subjects. For example, infected men are more expedient, jealous, dogmatic, and suspicious, whereas infected women are more conscientious, persistent, warm-hearted, outgoing, and moralistic than non-infected subjects (Flegr, 2007). Higher financial risk behavior has also been reported in *Toxoplasma*-infected individuals (Johnson et al., 2018). In a recent study, Borráz-León et al. (*2021a*) reported that *Toxoplasma*-infected men scored higher in psychoticism and interpersonal sensitivity than non-infected men. Still, no significant differences were found for women, regardless of their infection status. Taking together, these endocrinological, behavioral, and physical changes might influence the attractiveness and mating success of the hosts, which ultimately would benefit the spread of *T. gondii* to new bodies as this parasite could be sexually transmitted in several mammal species, including humans (*e.g*., Arantes et al., 2009; Dass et al., 2011; Flegr, Klapilová & Kaňková, 2014; Hlaváčová et al., 2020; Tong et al., 2021). For example, according to the multidimensional model of the fast-slow life history continuum (Del Giudice, 2014; 2017), elevated testosterone levels in infected men, in combination with changes in their personality traits, may promote the “seductive/creative” phenotype, which would be associated with higher mating success (Del Giudice, 2019). However, the research on this topic is still limited, and many questions remain unanswered.

Therefore, the present research had two main aims: (1) To compare differences between *Toxoplasma*-infected and non-infected subjects in several direct and indirect measures of a genetic condition, health, mating success, and parasitic resistance (*i.e*., self-perceived attractiveness, number of sexual partners, number of minor ailments, BMI, mate value, handgrip strength (HGS), facial FA, and the facial width-to-height ratio (fWHR)) (*e.g*., Scheib, Gangestad & Thornhill, 1999; Gallup & Fink, 2018; Borráz-León, Cerda-Molina & Mayagoitia-Novales, 2017; Luoto et al., 2021). (2) To investigate whether other people perceive differences in attractiveness and health between *Toxoplasma*-infected and non-infected subjects of both sexes. Since attractiveness evaluations may be modulated by raters’ characteristics such as self-perceived attractiveness, health, age, sex, relationship status, ethnicity (*e.g*., Feinberg et al., 2012; Borráz-León et al., 2014), and/or disgust sensitivity (*e.g*., Park, van Leeuwen & Stephen, 2012; Lee et al., 2015), we included these variables in the analyses.

For the first aim, we hypothesized that *Toxoplasma*-infected subjects might show higher self-perception of attractiveness, score higher in mate value, report a higher number of sexual partners and fewer minor ailments, have greater HGS in men (but less in women), lower facial FA, and higher fWHR in men (but lower in women); all these characteristics would facilitate the spread of *T. gondii* to new hosts. For the second aim, we hypothesized that the faces of *Toxoplasma*-infected men and women might be rated as more attractive and healthier than the faces of non-infected subjects. Furthermore, we hypothesized that lower disgust sensitivity scores (especially pathogen disgust) might mediate the attractiveness and health evaluations.

## Materials and Methods

### Ethical note

This study was approved by the Research and Ethics Committees of the National Institute of Psychiatry “Ramón de la Fuente Muñiz” (Project Number: NC 17076.0). All participants provided their written or online consent to participate in this study. The experiment was conducted according to the Declaration of Helsinki.

### Participants and *Toxoplasma* status

For testing the first hypothesis, we collected anthropometric and behavioral data from a sample of 213 healthy college students of the National Autonomous University of Mexico, Mexico (*n*_males_ = 108, *n*_females_ = 105) (mean age = 22.05 ± SD = 3.96) who were previously tested for the presence of specific *Toxoplasma* IgG (see Borráz-León et al. (2021a) for more details). From this sample, 35 subjects (22 men and 13 women) tested positive for *Toxoplasma* IgG, whereas 178 (86 men and 92 women) did not.

All participants received information about the aim of the study following the approval of the appropriate local ethics committee, signed an online letter of informed consent, and filled out a general demographic questionnaire with questions about age, body mass, height, marital status, number of minor ailments in the last year, and the number of sexual partners.

### Self-perceived attractiveness

A single question was used to measure self-perception of attractiveness (*i.e*., “How attractive do you consider yourself?”) with a 5-point Likert-scale (1 = low attractiveness, 3 = average attractiveness, 5 = high attractiveness) (Little et al., 2001; Borráz-León & Rantala, 2021).

### Mate value measurements

Mate value was measured using the self-report Mate Value Scale (MVS) (Edlund & Sagarin, 2014). This scale consists of four items (*e.g*., “Overall, how would you rate your level of desirability as a partner on the following scale?”) with a 7-point Likert-scale ranging from 1 = extremely undesirable to 7 = extremely desirable.

### Hand grip strength (HGS) measurements

HGS is a robust measure of overall muscular strength that is commonly used as a predictor of multiple health factors and fitness outcomes for both men and women (Gallup & Fink, 2018). HGS was measured in kilograms using a digital dynamometer (Baseline® 12-0286, USA). *Toxoplasma*-infected and non-infected participants were asked to perform a maximum force trial three times for their dominant hand. The HGS of each individual was calculated by averaging the result of the three force trials.

### Facial fluctuating asymmetry

A photograph of each participant’s face was taken using a Samsung NX1100, 20.3MPx digital camera from a distance of 2 m. The pictures were taken in the same natural light conditions. Participants were instructed to assume a neutral facial expression with their mouths closed. Before facial FA measurements, all pictures were horizontally aligned and scaled according to inter-pupillary distance (Grammer & Thornhill, 1994; Borráz-León, Rantala & Cerda-Molina, 2019). Facial FA was calculated from the Procrustes distances from 39 facial landmarks using the MorphoJ software (Klingenberg, 2011; see Sanchez-Pages & Turiegano (2010) and Muñoz-Reyes et al. (2012) for details about the technique employed to measure facial FA).

### Facial width-to-height ratio (fWHR)

As reported in Lewis, Lefevre & Bates (2012), fWHR was calculated by measuring the bizygomatic width (i.e., the maximum horizontal distance between the left and the right facial boundaries) and the upper-face height (*i.e*., the vertical distance between the highest point of the upper lip and the highest point of the eyelids). The fWHR was calculated as width divided by height using the same pictures used for facial FA measurements.

### Attractiveness and health perceptions of *Toxoplasma*-infected and non-infected subjects

**Figure 1:**
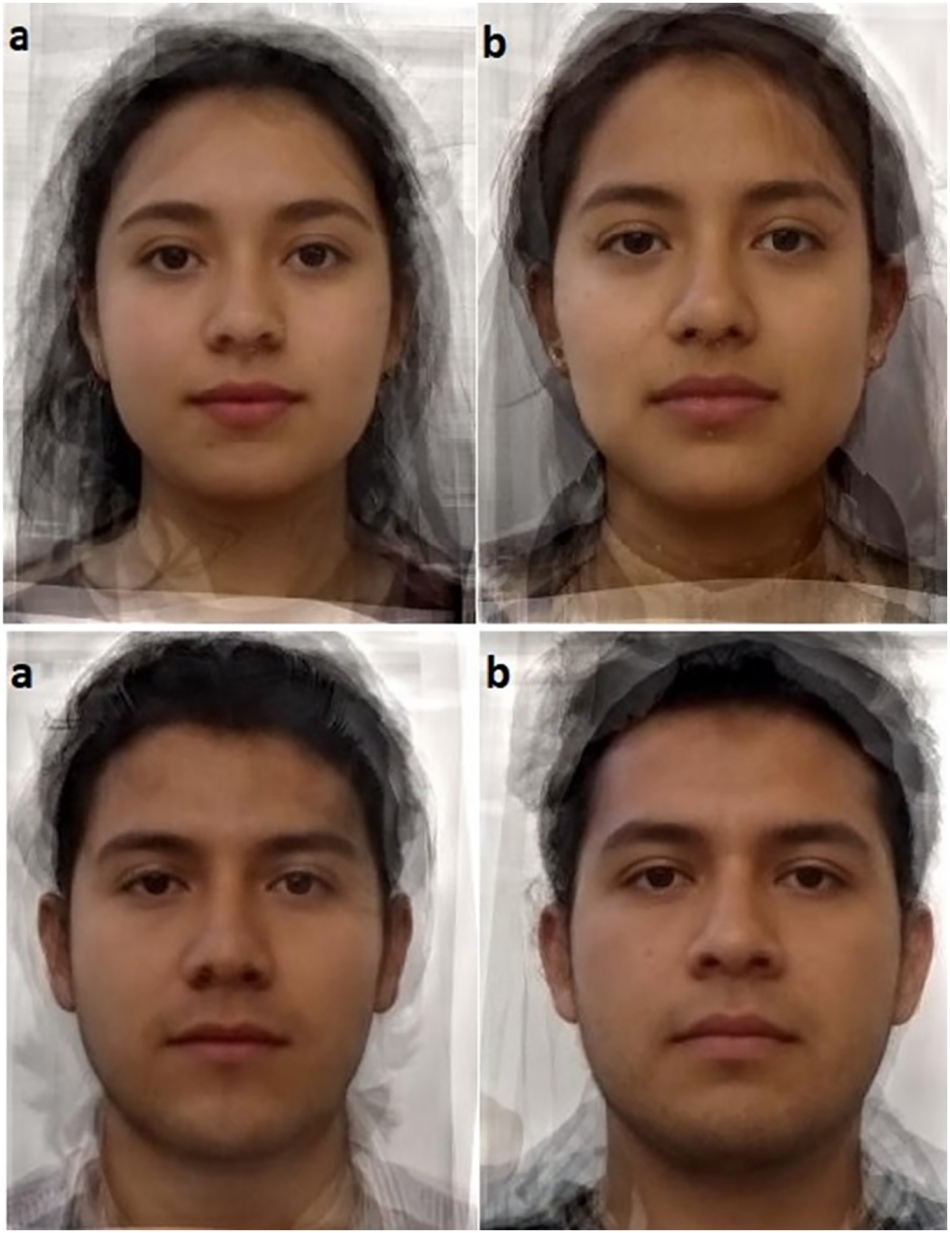
(A) Composite images of ten Toxoplasma-infected women and ten Toxoplasma-infected men, (B) composite images of ten non-infected women and ten non-infected men. Each picture represents the average face of men and women with and without *Toxoplasma* infection. Since each picture was created by merging photos of 10 individuals for each category, the faces shown in the pictures are not real individuals.

For testing the second hypothesis, facial pictures of *Toxoplasma*-infected and non-infected subjects were evaluated in sets of 20 pictures randomly organized each time (*i.e*., 10 pictures of *Toxoplasma*-infected subjects and 10 non-infected) by an independent group of 205 participants (hereinafter raters) (*n*_males_ = 59, *n*_females_ = 146) (mean age = 26.23 ± SD = 4.88), first for facial attractiveness and then for perceived health using a 10-point Likert-scale (1 = Very unattractive/unhealthy, 10 = Very attractive/healthy, respectively). In addition, raters evaluated the attractiveness and perceived health of four composite faces created with the Psychomorph software (Tiddeman, Perrett & Burt, 2001). These images represent the average face of a *Toxoplasma*-infected man (created from the faces of 10 infected men) and the average face of a *Toxoplasma*-infected woman (created from the faces of 10 infected women). The average faces of a non-infected man and a non-infected woman were also created (both of them created from the faces of 10 non-infected men and non-infected women, respectively) (Fig. 1). To avoid a bias in raters’ evaluations, we informed them about the purpose of the study after completing their participation.

Besides the general demographic questionnaire, raters answered the disgust sensitivity scale (Tybur, Lieberman & Griskevicius, 2009) that includes pathogen disgust, sexual disgust, and moral disgust to assess whether the disgust sensitivity of raters modulates the attractiveness and health perceptions of *Toxoplasma*-infected and non-infected subjects.

Demographic characteristics of raters were as follows: *Nationality*: Finnish (61.5%), French (7.3%), English (4.9%), and Mexican (26.3%). *Ethnicity*: Caucasian (70.7%), Hispanic/Latino (27.3%), and Other (2.0%). *Relationship status*: In a relationship (50.2%), single (41.0%), other (8.8%).

### Statistical analyses

Since our data did not meet the normality criteria after being log-transformed (Shapiro-Wilk Test: *p* < 0.05 in all cases), we ran non-parametric tests for testing the first hypothesis: Independent Mann-Whitney *U* tests were used to assess potential sex differences and differences between *Toxoplasma*-infected and non-infected subjects.

For testing the second hypothesis, we used *t*-tests to look for differences between *Toxoplasma*-infected *vs*. non-infected subjects after log-transforming the data for increasing normality (*e.g*., Luoto et al., 2021). Then, we used a Multivariate Analysis of Covariance (MANCOVA) to analyze the effect of the characteristics of raters (*i.e*., age, BMI, self-perceived attractiveness, self-rated health, sex, ethnicity, relationship status, and the three categories of disgust sensitivity) on the given evaluations of attractiveness and perceived health in both *Toxoplasma*-infected and non-infected subjects. The threshold for statistical significance in all analyses was set at *p* ≤ 0.05. The data were analyzed using SPSS version 25 (SPSS Inc., Chicago, IL, USA).

## Results

### Sex differences

Men had higher fWHR, were older, taller, had higher body mass, BMI, scored higher in HGS, and reported a higher number of sexual partners than women. No significant sex differences were found for the other variables (Table 1).

**Table 1:** Sex differences (*n* = 213). Sex differences in all the included variables of this research.

	Men (*n* = 108)	Women (*n* = 105)				
	Mean (*SD*)	Mean (*SD*)	*p*	Hedges’ *g*	95% CI for Hedges’ *g*	
					Lower	Upper
Facial FA	0.02 (0.01)	0.02 (0.01)	0.452	−0.08	−0.18	0.35
fWHR	2.11 (0.18)	2.06 (0.15)	0.024*	−0.38	−0.66	−0.11
Age	22.92 (4.53)	21.15 (3.05)	<0.001***	−0.45	−0.72	−0.18
Body mass	73.18 (13.27)	59.27 (10.49)	<0.001***	−1.16	−1.45	−0.87
BMI	24.63 (4.11)	23.17 (3.60)	0.009**	−0.37	−0.64	−0.10
Height	1.72 (0.06)	1.59 (0.07)	<0.001***	−1.99	−2.32	−1.66
Self-perceived attractiveness	3.29 (0.84)	3.44 (0.86)	0.193	0.17	−0.09	0.44
Number of sexual partners	6.26 (6.51)	3.77 (4.11)	0.007**	−0.45	−0.72	−0.18
Previous ailments	0.81 (0.66)	0.98 (0.67)	0.059	0.25	−0.01	0.52
Mate value	19.47 (4.28)	20.16 (4.24)	0.233	0.16	−0.10	0.43
Hand-grip strength	39.73 (8.08)	24.49 (4.86)	<0.001***	−2.27	−2.62	−1.93

**Notes:**

* *p* < 0.05.

** *p* < 0.01.

*** *p* < 0.001.

FA: Fluctuating asymmetry; fWHR: Facial width-to-height ratio; BMI: Body mass index.

### Differences according to *Toxoplasma* *gondii* status and sex

**Figure 2:**
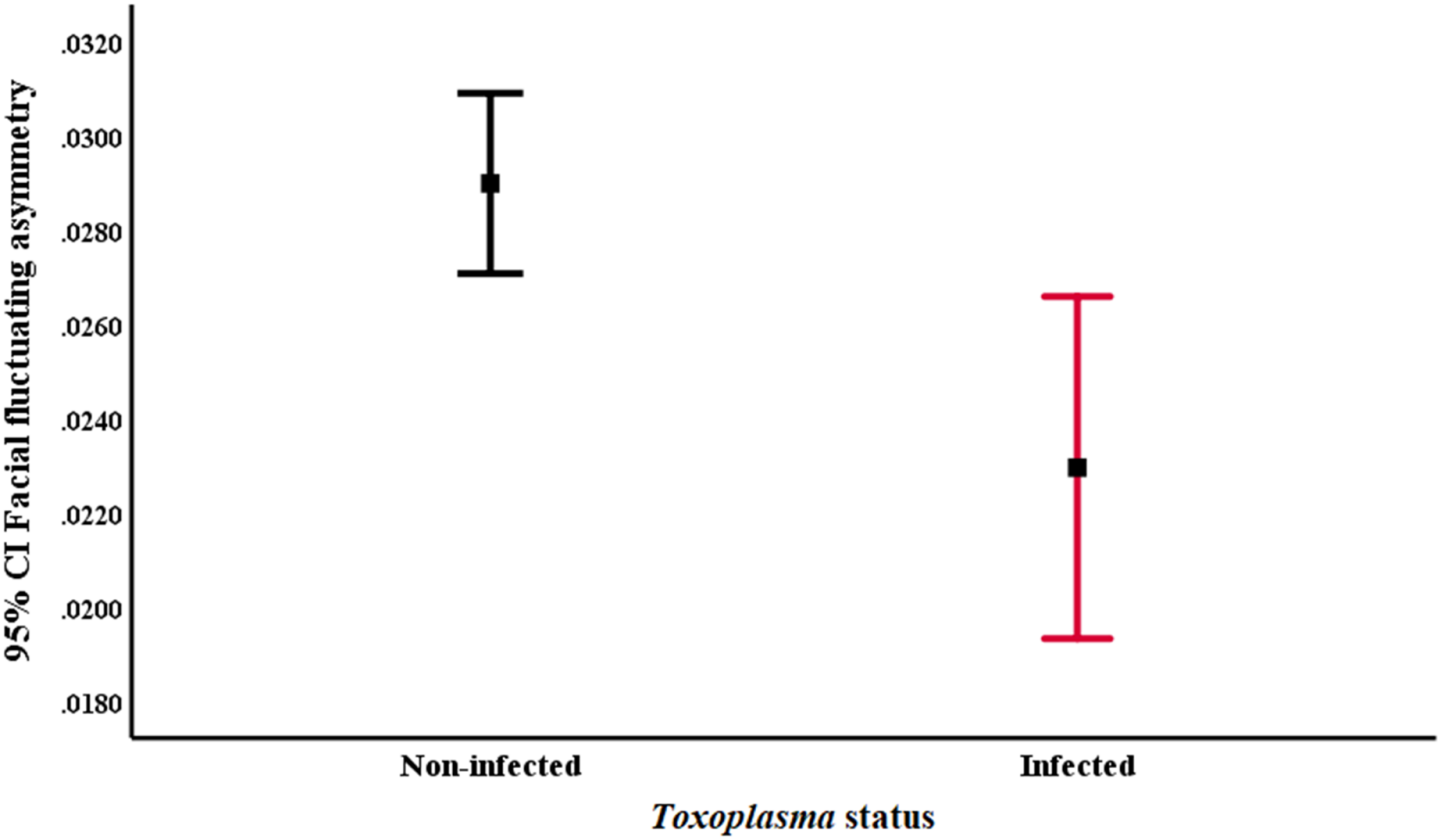
*Toxoplasma*-infected subjects have lower facial fluctuating asymmetry than non-infected ones (*p* = 0.006; Hedge’s *g* = 0.51; 95% CI = [0.14 to 0.87]). The figure represents the 95% CI of facial fluctuating asymmetry between *Toxoplasma*-infected and non-infected subjects.

*Toxoplasma*-infected subjects had significantly lower facial FA than non-infected ones (*p* = 0.006; Hedge’s *g* = 0.51; 95% CI = [0.14 to 0.87]) (Fig. 2). No significant differences were found for the other variables (see Table 2). Broken out by sex, the analyses showed that *Toxoplasma*-infected men had lower levels of facial FA than non-infected ones (*p* = 0.047; Hedge’s *g* = 0.39; 95% CI = [−0.07 to 0.86]), whereas *Toxoplasma*-infected women had lower body mass (*p* = 0.002; Hedge’s *g* = 0.83; 95% CI = [0.24 to 1.42]), and lower BMI (*p* = 0.015; Hedge’s *g* = 0.65; 95% CI = [0.07 to 1.24]) than non-infected women. There was also a tendency for infected women to have lower facial FA (*p* = 0.055; Hedge’s *g* = 0.63; 95% CI = [0.01 to 1.19]), to report higher self-perceived attractiveness (*p* = 0.053; Hedge’s *g* = −0.65; 95% CI = [−1.24 to −0.06]), and a higher number of sexual partners (*p* = 0.064; Hedge’s *g* = −0.53; 95% CI = [−1.12 to 0.04]), than non-infected women. No significant results were found for the other variables (see Table 2).

**Table 2:** Differences according to *Toxoplasma* status (*n* = 213). This table shows the differences between *Toxoplasma*-infected and non-infected ones regarding all the studied variables.

	*Toxoplasma*-infected subjects (*n* = 35)	Non-infected subjects (*n* = 178)				
	Mean (*SD*)	Mean (*SD*)	*p*	Hedges’ *g*	95 % CI for Hedges’ *g*	
					Lower	Upper
Facial FA	0.02 (0.01)	0.02 (0.01)	0.006**	0.51	0.14	0.87
fWHR	2.08 (0.19)	2.09 (0.17)	0.798	0.05	−0.30	0.42
Age	23.17 (4.88)	21.83 (3.73)	0.252	−0.34	−0.70	0.02
Body mass	66.74 (15.86)	66.24 (13.45)	0.975	−0.03	−0.39	0.32
BMI	24.16 (5.00)	23.86 (3.70)	0.762	−0.07	−0.43	0.28
Height	1.65 (0.09)	1.66 (0.09)	0.987	0.11	−0.25	0.47
Self-perceived attractiveness	3.46 (0.98)	3.34 (0.83)	0.478	−0.14	−0.50	0.22
Number of sexual partners	6.11 (6.29)	4.82 (5.44)	0.200	−0.23	−0.59	0.13
Previous ailments	0.97 (0.66)	0.88 (0.67)	0.437	−0.13	−0.49	0.22
Mate value	19.54 (4.06)	19.87 (4.31)	0.701	0.07	−0.28	0.44
Hand-grip strength	34.28 (11.23)	31.81 (9.89)	0.219	−0.24	−0.60	0.11
	*Toxoplasma*-infected men (*n* = 22)	Non-infected men (*n* = 86)				
Facial FA	0.02 (0.01)	0.02 (0.01)	0.047*	0.39	−0.07	0.86
fWHR	2.12 (0.22)	2.11 (0.28)	0.948	−0.37	−0.84	0.09
Age	24.36 (5.25)	22.55 (4.29)	0.188	−0.40	−0.87	0.06
Body mass	75.55 (13.09)	72.58 (13.32)	0.344	−0.22	−0.69	0.24
BMI	25.95 (5.40)	24.29 (3.68)	0.204	−0.40	−0.87	0.06
Height	1.71 (0.06)	1.72 (0.06)	0.392	0.16	−0.30	0.63
Self-perceived attractiveness	3.18 (0.95)	3.31 (0.81)	0.636	0.15	−0.31	0.62
Number of sexual partners	6.36 (6.23)	6.23 (6.62)	0.924	−0.02	−0.48	0.44
Previous ailments	0.86 (0.64)	0.79 (0.67)	0.612	−0.10	−0.57	0.36
Mate value	19.36 (3.94)	19.50 (4.39)	0.963	0.03	−0.43	0.50
Hand-grip strength	40.75 (8.11)	39.47 (8.09)	0.471	−0.15	−0.62	0.31
	*Toxoplasma*-infected women (*n* = 13)	Non-infected women (*n* = 92)				
Facial FA	0.02 (0.00)	0.02 (0.01)	0.055	0.63	0.01	1.19
fWHR	2.02 (0.12)	2.06 (0.16)	0.459	0.25	−0.32	0.83
Age	21.15 (3.50)	21.15 (3.00)	0.898	0.00	−0.58	0.58
Body mass	51.85 (5.47)	60.32 (10.62)	0.002**	0.83	0.24	1.42
BMI	21.13 (2.01)	23.46 (3.69)	0.015*	0.65	0.07	1.24
Height	1.56 (0.05)	1.60 (0.07)	0.143	0.58	0.00	1.17
Self-perceived attractiveness	3.92 (0.86)	3.37 (0.84)	0.053	−0.65	−1.24	−0.06
Number of sexual partners	5.69 (6.62)	3.50 (3.60)	0.064	−0.53	−1.12	0.04
Previous ailments	1.15 (0.68)	0.96 (0.67)	0.327	−0.28	−0.86	0.29
Mate value	19.85 (4.41)	20.21 (4.23)	0.815	0.08	−0.49	0.66
Hand-grip strength	23.33 (5.96)	24.65 (4.70)	0.414	0.27	−0.31	0.85

**Notes:**

* *p* < 0.05.

** *p* < 0.01.

FA: Fluctuating asymmetry; fWHR: Facial width-to-height ratio; BMI: Body mass index.

### Attractiveness and health perceptions in *Toxoplasma*-infected and non-infected subjects

**Figure 3:**
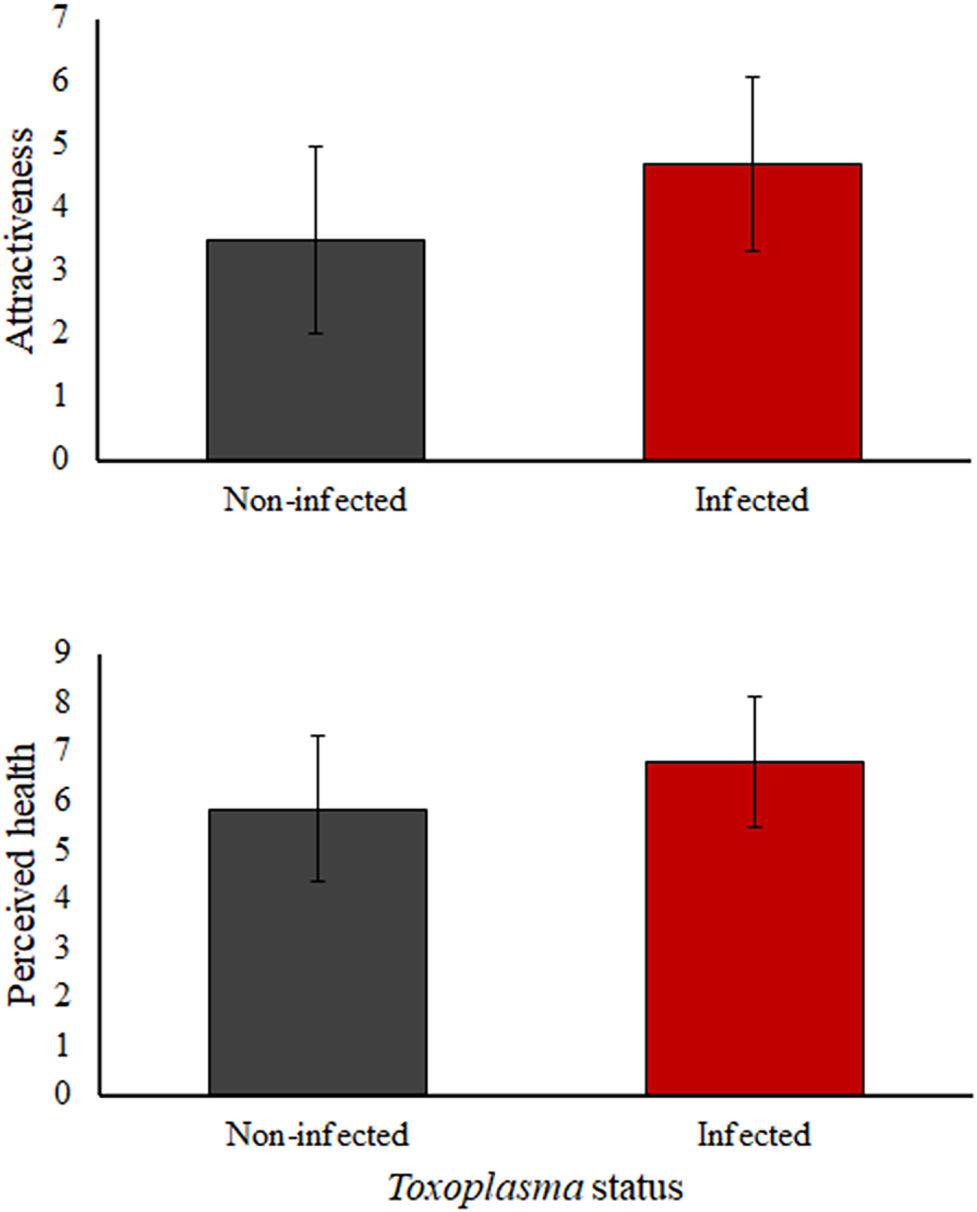
*Toxoplasma*-infected subjects from both sexes were rated as more attractive (*t* = −8.398, *p* < 0.001) and healthier (*t* = −6.659, *p* < 0.001) than non-infected ones. This figure represents the mean and the standard deviation of attractiveness (above) and health (below) of *Toxoplasma*-infected and non-infected subjects.

*Toxoplasma*-infected subjects (both men and women) were rated as significantly more attractive (*t* = −8.398, *p* < 0.001) and healthier (*t* = −6.659, *p* < 0.001) than non-infected ones (Fig. 3). No significant sex differences were found for attractiveness and health evaluations of *Toxoplasma*-infected and non-infected subjects (*t* = −0.871, *p* = 0.378; *t* = 0.275, *p* = 0.784 respectively).

Even though we found that female raters tended to give higher scores of health than male raters (*t* = −2.796, *p* = 0.005), both men and women, analyzed separately, rated as significantly healthier *Toxoplasma*-infected subjects than non-infected ones (men only: *t* = −3.364, *p* = 0.001; women only: *t* = −5.842, *p* < 0.001). No sex differences were found for the attractiveness scores given (*t* = 1.703, *p* = 0.089).

Significant multivariate effects of pathogen disgust (Wilks λ: 0.94, *F* = 2.681, *df* = 4/180, *p* = 0.033, *η*^*2*^ = 0.056, Observed power = 0.74), and a tendency of BMI (Wilks λ: 0.95, *F* = 2.203, *df* = 4/180, *p* = 0.070, *η*^*2*^ = 0.047, Observed power = 0.64) were found. No significant multivariate effects were found for the other studied variables (see Table S1).

Univariate effects were found for BMI on the evaluation of health perceptions in *Toxoplasma*-infected (*F* = 8.40, *df* = 1/183, *p* = 0.004) and non-infected subjects (*F* = 6.69, *df* = 1/183, *p* = 0.010), for pathogen disgust on attractiveness perceptions in *Toxoplasma*-infected (*F* = 9.31, *df* = 1/183, *p* = 0.003) and non-infected subjects (*F* = 8.65, *df* = 1/183, *p* = 0.004), and for pathogen disgust on health perceptions in non-infected subjects (*F* = 3.94, *df* = 1/183, *p* = 0.048).

In addition, there were tendencies of raters’ self-perceived health on health perceptions in *Toxoplasma*-infected subjects (*F* = 3.56, *df* = 1/183, *p* = 0.061), of pathogen disgust on health perception in *Toxoplasma*-infected subjects (*F* = 3.41, *df* = 1/183, *p* = 0.066), and of sexual disgust on attractiveness perceptions in *Toxoplasma*-infected subjects (*F* = 3.34, *df* = 1/183, *p* = 0.069); non-significant results were found for the other studied variables (see Table S2).

## Discussion

For the first hypothesis, we found that *Toxoplasma*-infected subjects had lower facial FA than non-infected ones. Broken out by sex, we found that *Toxoplasma*-infected men had lower facial FA than non-infected men, whereas *Toxoplasma*-infected women had lower body mass, lower BMI, and a tendency to lower facial FA, higher self-perceived attractiveness, and a higher number of sexual partners than non-infected ones. Previous studies have reported positive associations between symmetrical traits, health, good genes, attractiveness, and parasitic resistance both in humans and non-human animals (*e.g*., Møller, 1992; Gangestad, Thornhill & Yeo, 1994; Polak, 1997; Borráz-León et al., 2021b; Luoto et al., 2021). Thus, one possible explanation for our results is that highly symmetrical subjects can successfully afford the physiological costs related to parasitism, which supports the hypothesis that highly symmetrical features are honest signals of good health (Borráz-León, Cerda-Molina & Mayagoitia-Novales, 2017; Luoto et al., 2021, but see Pound et al., 2014). Another possibility is that *T. gondii* infection may produce changes in facial symmetry of its hosts through changes in endocrinological variables such as testosterone levels; this hypothesis is consistent with the finding that *Toxoplasma*-infected men have higher testosterone levels (Flegr, Lindová & Kodym, 2008; Borráz-León et al., 2021a) and *Toxoplasma*-infected women have lower testosterone levels than non-infected subjects (Flegr, Lindová & Kodym, 2008), as well as with some data showing that body symmetry can change due to hormonal fluctuations across the menstrual cycle (Scutt & Manning, 1996; but see Marcinkowska & Holzleitner, 2020). These changes, both in the endocrinology system and in facial symmetry, would ultimately benefit the spread of the parasite by increasing the attractiveness of its hosts through increasing their facial symmetry. This hypothesis is also consistent with previous results showing that *Toxoplasma*-infected male rats have higher testosterone levels and are preferred as sexual partners by non-infected females (Dass et al., 2011), and with positive relationships between high testosterone levels, attractiveness, and immune responses in men (Rantala et al., 2012; Luoto et al., 2021). However, this hypothesis must be empirically tested further since other studies have found no direct associations between testosterone levels and facial symmetry (*e.g*., Borráz-León et al., 2014).

Regarding the lower body mass and the lower BMI of *Toxoplasma*-infected women, it is important to highlight that the values of both physical measurements are within what is considered “normal” and healthy (Brown et al., 2016). These results support the view that *T. gondii* might also increase the metabolic rate of its hosts which would influence their health and attractiveness perceptions. The tendency for reporting higher self-perceptions of attractiveness and a higher number of sexual partners among *Toxoplasma*-infected women is consistent with this explanation since the acquisition of sexual partners through more active mate-seeking, short-term sociosexual orientation, and higher attractiveness would facilitate the spread of *T. gondii* to new hosts. Another possibility is that *Toxoplasma*-infected subjects from both sexes are showing more attractive characteristics as a terminal investment strategy (*e.g*., Fessler et al., 2005). Nevertheless, this hypothesis is less plausible since there were no significant age differences between *Toxoplasma*-infected and non-infected subjects.

Regarding the second hypothesis, we found that other-perceptions of attractiveness and health are higher for *Toxoplasma*-infected subjects than for non-infected ones, independent of their sex. Moreover, the BMI and pathogen disgust of raters modulated the attractiveness and health perceptions of infected and non-infected subjects indistinctly. Interestingly, there also was a tendency for raters who had lower pathogen disgust and higher self-rated health to evaluate the faces of *Toxoplasma*-infected subjects as healthier than the faces of non-infected ones. Likewise, those raters with lower sexual disgust also tended to evaluate *Toxoplasma*-infected subjects as more attractive than non-infected ones. These results support and extend previous literature showing that, for example, *Toxoplasma*-infected male rats are preferred as sexual partners by females (Dass et al., 2011). Thus, it is possible that the same effect reported for *Toxoplasma*-infected male rats is present in *Toxoplasma*-infected humans. This effect may be modulated by phenotypic characteristics of potential mates such as disgust sensitivity and self-perceived health (*e.g*., Park, van Leeuwen & Stephen, 2012).

At this point, it is important to highlight that even though between 30% and 80% of the global population may be infected with *T. gondii* (*e.g*., Flegr, 2007; Johnson & Johnson, 2020), with transmission routes including contact with cat feces, contaminated food or water, or sexual intercourse with an infected person (Flegr, 2007; Flegr & Horáček, 2019; Tong et al., 2021), only a meager percentage of subjects (mainly those who are immunosuppressed) may develop severe complications associated with the infection (Johnson & Johnson, 2020). Likewise, a low percentage of infected subjects may develop mental disorders such as schizophrenia, obsessive-compulsive disorder, or suicidal tendencies (Flegr & Horáček, 2019; Torrey & Yolken, 2019; Nayeri, Sarvi & Daryani, 2022). However, it seems that exposure to previous traumatic experiences, chronic stress, genetic predisposition, drugs abuse, as well as *Toxoplasma* and other pathogen infections (*e.g*., Cytomegalovirus, herpes, *Clamydia spitacci*, *Treponema pallidum*) are all involved in the development of these mental disorders (MJ Rantala et al., 2021, unpublished data). In immunocompetent subjects, *Toxoplasma* infection may cause mild disease and usually turns into life-long latent toxoplasmosis being clinically asymptomatic (Roberts et al., 2001). Therefore, it is possible that the apparently non-pathological and potentially beneficial interactions between *T. gondii* and some of its intermediate hosts, such as rats and humans are the result of co-evolutionary strategies that benefit, or at least do not harm, the fitness of both the parasite and the host.

Some examples of parasites that manipulate the appearance and behavior of their hosts to increases their attractiveness have been previously described in nature. For example, female moths of *Helicoverpa zea* infected with gonad-specific virus (GSV) or Hz-2V virus produce three to seven times more sex pheromone than non-infected females which could increase their sexual attractiveness (Burand et al., 2005). Likewise, water snails (*Potamopyrugus antipodarum*) infected with a trematode (*Microphallus* sp.) enhance their sexual attractiveness measured by an increase in the number of mating events and the total number of different mating partners (Soper et al., 2014). Even though reports about host manipulation by sexually transmitted pathogens and the potential benefits these pathogens could gain remain scarce in humans (*e.g*., Heil, 2016). The present study offers novel evidence supporting the idea that some sexually transmitted parasites such as *T. gondii* (*e.g*., Hlaváčová et al., 2020; Tong et al., 2021), may produce changes in the appearance and behavior of the human host, either as a by-product of the infection or as the result of the manipulation of the parasite to increase its spread to new hosts.

### Limitations and future directions

The small sample size of the present study may have been an important limiting factor for finding statistical differences in the other studied phenotypic traits. Therefore, further studies with larger sample sizes must be carried out to confirm or reject the hypotheses tested in this study. Another limitation is that in the present study, physiological, immunological, and/or genetic markers associated with health were not directly measured. Although previous research has shown the advantages of using phenotypic over genotypic measurements of immune function and health (*e.g*., Luoto et al., 2021), future research must take the present limitations into account to offer a more complete explanation about how *T. gondii* may manipulate hosts’ behavior, morphology, and/or physiology.

Since reports that *T. gondii* can be a sexually transmitted parasite in humans are relatively recent (*e.g*., Flegr, Klapilová & Kaňková, 2014; Hlaváčová et al., 2020; Kaňková, Hlaváčová & Flegr, 2020; Tong et al., 2021), many questions and hypotheses still need to be properly formulated and tested in further studies. In this study, we provided the first evidence that *Toxoplasma*-infected individuals are more symmetrical and are perceived as more attractive and healthier than non-infected ones. Thus, further research can investigate, for example, the effect of *Toxoplasma*-status of raters on sociosexual orientation characteristics. It is possible that *Toxoplasma*-infected individuals may rate more positively the faces of potential mates which would benefit the spread of *T. gondii* by making them less *choosy* and probably exhibiting a short-term over a long-term sociosexual orientation. The effect of the menstrual cycle phase is another variable that have to be further studied. It could be possible that *Toxoplasma*-infected women who are in the fertile phase of the menstrual cycle, rate even more positively the faces of potential mates than *Toxoplasma*-infected women who are in other phase of the menstrual cycle, since changes in attractiveness perceptions during the menstrual cycle have been previously reported (*e.g*., Gangestad et al., 2007; Jones et al., 2008). Other future studies can look for differences in ejaculate quality between *Toxoplasma*-infected and non-infected men. Since *T. gondii* infection produces changes in testosterone levels, it is feasible that these changes may also affect some ejaculate parameters such as motility, sperm velocity, or ejaculate volume. In general, if the premises of the manipulation hypothesis by *T. gondii* are correct, such effects would increase its transmission rate to new hosts. Further research is also needed to study potential metabolic and physiological costs associated with *Toxoplasma* infection. For example, it is likely that the phenotypic changes of *Toxoplasma*-infected subjects such as lower facial FA, higher testosterone levels, and high-risk behaviors reported in this and other studies (Flegr, 2007; Johnson et al., 2018; Borráz-León et al., 2021a), may impose physiological costs to the host such as shortened life, reduced life quality during late adulthood, or increased predisposition to develop organic (*e.g*., hearth and pulmonary diseases) and mental diseases (*e.g*., depression, schizophrenia, bipolar disorder). Therefore, covariation between attractiveness, facial symmetry, changes in sex hormones and neurotransmitters, and the expression of physical and psychopathological symptoms, might be expected in genetically predisposed individuals. In the aggregate these research suggestions can shed light on the effects of *Toxoplasma* infection in human sociosexual and health domains.

## Conclusions

*T. gondii* is a parasite that can potentially affect the physiological and behavioral characteristics of its intermediate hosts (Poulin, 2010; Del Giudice, 2019). Some of these changes may increase the hosts’ mating success (Dass et al., 2011) and therefore represent transmission-related benefits for the parasite (Brüne, 2019). The present study offers novel evidence about that *T. gondii* infection may also be associated with phenotypic changes in infected humans (*e.g*., low facial FA in men and women, and low body mass and BMI in women), as well as with a better evaluation of their attractiveness and health. From an evolutionary point of view, such phenotypic changes may be either a by-product of the infection or the result of the manipulation exerted by *T. gondii* (Del Giudice, 2019). In any case, the observed phenotypic changes in *Toxoplasma*-infected subjects may represent transmission related benefits for the parasite as *T. gondii* can be sexually transmitted (Hlaváčová et al., 2020; Tong et al., 2021). This research lays out the foundation for the study of the potential manipulation processes of the human host by sexually transmitted pathogens and parasites.

## Supplemental Information

10.7717/peerj.13122/supp-1 Supplemental Information 1 Raw data for hypothesis 1. All the raw data we used for testing the first hypothesis of our study.

10.7717/peerj.13122/supp-2 Supplemental Information 2 Raw data for hypothesis 2. All the raw data we used for testing the second hypothesis of our study.

10.7717/peerj.13122/supp-3 Supplemental Information 3 Multivariate effects of the MANCOVA.

10.7717/peerj.13122/supp-4 Supplemental Information 4 Univariate effects on attractiveness and health in *Toxoplasma*-infected and non-infected subjects. The univariate effects of each one of the studied variables on other-attractiveness and other-health evaluations.

## Additional Information and Declarations

### Data Availability

The following information was supplied regarding data availability: The raw data is available in the Supplemental Files.
