# Supplementary material for: Are Toxoplasma-infected subjects more attractive, symmetrical, or healthier than non-infected ones? Evidence from subjective and objective measurements
Source: PeerJ. 2022 Mar 25;10:e13122. doi: 10.7717/peerj.13122 (PMC8958965; doi:10.7717/peerj.13122)
Supplement: Supplemental Information 3 [file peerj-10-13122-s003.docx]

**Table S1.** Multivariate effects.

| **Source** | **Wilks λ** | ***F*** | ***df*** | ***p*** | ***η^2^*** | **OP** |
| --- | --- | --- | --- | --- | --- | --- |
| **Intersection** | 0.836 | 8.802 | 4/180 | <0.001*** | 0.164 | 0.999 |
| **Age** | 0.972 | 1.289 | 4/180 | 0.276 | 0.028 | 0.398 |
| **BMI** | 0.953 | 2.203 | 4/180 | 0.070 | 0.047 | 0.640 |
| **Self-perceived attractiveness** | 0.983 | 0.777 | 4/180 | 0.541 | 0.017 | 0.246 |
| **Self-rated health** | 0.976 | 1.122 | 4/180 | 0.348 | 0.024 | 0.348 |
| **Pathogen disgust** | 0.944 | 2.681 | 4/180 | 0.033* | 0.056 | 0.737 |
| **Sexual disgust** | 0.958 | 1.988 | 4/180 | 0.098 | 0.042 | 0.589 |
| **Moral disgust** | 0.960 | 1.898 | 4/180 | 0.113 | 0.040 | 0.566 |
| **Sex** | 0.976 | 1.126 | 4/180 | 0.346 | 0.024 | 0.350 |
| **Ethnicity** | 0.960 | 0.922 | 8/360 | 0.498 | 0.020 | 0.430 |
| **Marital status** | 0.961 | 0.897 | 8/360 | 0.519 | 0.020 | 0.419 |
| **Sex*Ethnicity** | 0.993 | 0.304 | 4/180 | 0.875 | 0.007 | 0.117 |
| **Sex*Marital status** | 0.965 | 0.803 | 8/360 | 0.600 | 0.018 | 0.374 |
| **Ethnicity*Marital status** | 0.975 | 0.282 | 16/550 | 0.998 | 0.006 | 0.150 |
| **Sex*Ethnicity*Marital status** | 0.988 | 0.281 | 8/360 | 0.972 | 0.006 | 0.141 |

BMI = body mass index; OP = observed power; * *p* < 0.05; ** *p* < 0.01; *** *p* < 0.001
