# Supplementary material for: Are Toxoplasma-infected subjects more attractive, symmetrical, or healthier than non-infected ones? Evidence from subjective and objective measurements
Source: PeerJ. 2022 Mar 25;10:e13122. doi: 10.7717/peerj.13122 (PMC8958965; doi:10.7717/peerj.13122)
Supplement: Supplemental Information 4 — The univariate effects of each one of the studied variables on other-attractiveness and other-health evaluations. [file peerj-10-13122-s004.docx]

**Table S2.** Univariate effects on attractiveness and health in *Toxoplasma*-infected and non-infected subjects.

|  | **Dependent Variables** | ***F*** | ***df*** | ***p*** | ***η^2^*** | **OP** |
| --- | --- | --- | --- | --- | --- | --- |
| **Intersection** | AT | 15.922 | 1/183 | <0.001*** | 0.080 | 0.978 |
|  | AN | 12.230 | 1/183 | 0.001*** | 0.063 | 0.936 |
|  | HT | 31.789 | 1/183 | <0.001*** | 0.148 | 1.000 |
|  | HN | 21.227 | 1/183 | <0.001*** | 0.104 | 0.996 |
| **Age** | AT | 1.398 | 1/183 | 0.239 | 0.008 | 0.217 |
|  | AN | 1.959 | 1/183 | 0.163 | 0.011 | 0.286 |
|  | HT | 1.258 | 1/183 | 0.263 | 0.007 | 0.200 |
|  | HN | 1.002 | 1/183 | 0.318 | 0.005 | 0.169 |
| **BMI** | AT | 0.399 | 1/183 | 0.528 | 0.002 | 0.096 |
|  | AN | 0.264 | 1/183 | 0.608 | 0.001 | 0.080 |
|  | HT | 8.403 | 1/183 | 0.004** | 0.044 | 0.822 |
|  | HN | 6.695 | 1/183 | 0.010** | 0.035 | 0.730 |
| **Self-perceived attractiveness** | AT | 0.023 | 1/183 | 0.880 | 0.035 | 0.987 |
|  | AN | 0.814 | 1/183 | 0.368 | 0.000 | 0.720 |
|  | HT | 0.099 | 1/183 | 0.754 | 0.004 | 0.630 |
|  | HN | 0.793 | 1/183 | 0.374 | 0.001 | 0,916 |
| **Self-rated health** | AT | 0.063 | 1/183 | 0.803 | 0.000 | 0.057 |
|  | AN | 0.039 | 1/183 | 0.843 | 0.000 | 0.054 |
|  | HT | 3.564 | 1/183 | 0.061 | 0.019 | 0.466 |
|  | HN | 2.122 | 1/183 | 0.147 | 0.011 | 0.305 |
| **Pathogen disgust** | AT | 9.319 | 1/183 | 0.003** | 0.048 | 0.859 |
|  | AN | 8.655 | 1/183 | 0.004** | 0.045 | 0.833 |
|  | HT | 3.411 | 1/183 | 0.066 | 0.018 | 0.451 |
|  | HN | 3.949 | 1/183 | 0.048* | 0.021 | 0.507 |
| **Sexual disgust** | AT | 3.346 | 1/183 | 0.069 | 0.018 | 0.444 |
|  | AN | 0.372 | 1/183 | 0.543 | 0.002 | 0.093 |
|  | HT | 2.075 | 1/183 | 0.151 | 0.011 | 0.299 |
|  | HN | 1.027 | 1/183 | 0.312 | 0.006 | 0.172 |
| **Moral disgust** | AT | 0.130 | 1/183 | 0.719 | 0.001 | 0.065 |
|  | AN | 0.004 | 1/183 | 0.949 | 0.000 | 0.050 |
|  | HT | 0.225 | 1/183 | 0.636 | 0.001 | 0.076 |
|  | HN | 1.742 | 1/183 | 0.189 | 0.009 | 0.259 |
| **Sex** | AT | 2.775 | 1/183 | 0.097 | 0.015 | 0.381 |
|  | AN | 0.877 | 1/183 | 0.350 | 0.005 | 0.154 |
|  | HT | 2.181 | 1/183 | 0.141 | 0.012 | 0.312 |
|  | HN | 0.905 | 1/183 | 0.343 | 0.005 | 0.157 |
| **Ethnicity** | AT | 0.621 | 2/183 | 0.539 | 0.007 | 0.153 |
|  | AN | 0.844 | 2/183 | 0.432 | 0.009 | 0.193 |
|  | HT | 0.118 | 2/183 | 0.889 | 0.001 | 0.068 |
|  | HN | 0.372 | 2/183 | 0.690 | 0.004 | 0.109 |
| **Relationship status** | AT | 2.174 | 2/183 | 0.117 | 0.023 | 0.441 |
|  | AN | 1.494 | 2/183 | 0.227 | 0.016 | 0.316 |
|  | HT | 0.952 | 2/183 | 0.388 | 0.010 | 0.213 |
|  | HN | 0.514 | 2/183 | 0.599 | 0.006 | 0.134 |
| **Sex*Ethnicity** | AT | 0.106 | 1/183 | 0.746 | 0.001 | 0.062 |
|  | AN | 0.444 | 1/183 | 0.506 | 0.002 | 0.102 |
|  | HT | 0.088 | 1/183 | 0.767 | 0.000 | 0.060 |
|  | HN | 0.120 | 1/183 | 0.729 | 0.001 | 0.064 |
| **Sex*Relationship status** | AT | 0.831 | 2/183 | 0.437 | 0.009 | 0.191 |
|  | AN | 1.109 | 2/183 | 0.332 | 0.012 | 0.243 |
|  | HT | 0.108 | 2/183 | 0.898 | 0.001 | 0.066 |
|  | HN | 0.270 | 2/183 | 0.763 | 0.003 | 0.092 |
| **Ethnicity*Relationship status** | AT | 0.134 | 4/183 | 0.970 | 0.003 | 0.077 |
|  | AN | 0.164 | 4/183 | 0.956 | 0.004 | 0.084 |
|  | HT | 0.445 | 4/183 | 0.776 | 0.010 | 0.153 |
|  | HN | 0.532 | 4/183 | 0.713 | 0.011 | 0.177 |
| **Sex*Ethnicity*Relationship status** | AT | 0.158 | 2/183 | 0.854 | 0.002 | 0.074 |
|  | AN | 0.321 | 2/183 | 0.726 | 0.003 | 0.101 |
|  | HT | 0.472 | 2/183 | 0.625 | 0.005 | 0.126 |
|  | HN | 0.324 | 2/183 | 0.724 | 0.004 | 0.101 |

AT: Attractiveness of *Toxoplasma*-infected subjects: AN: Attractiveness of non-infected subjects; HT: Health of *Toxoplasma*-infected subjects; HN: Health of non-infected subjects; **p* < 0.05; ***p* < 0.01; ****p* < 0.001
